# Social trust and emotional health in rural older adults in China: the mediating and moderating role of subjective well-being and subjective social status

**DOI:** 10.1186/s12889-021-10617-y

**Published:** 2021-03-20

**Authors:** Hongsheng Chen, Zhenjun Zhu

**Affiliations:** 1 School of Architecture and Urban Planning, Shenzhen University, Shenzhen, 518060 Guangdong China; 2 College of Automobile and Traffic Engineering, Nanjing Forestry University, No. 159 Longpan Road, Xuanwu District, Nanjing, 210037 China

**Keywords:** Social trust, Emotional health, Family, Friends, Neighbours, Subjective well-being, Subjective social status

## Abstract

**Background:**

China is becoming an aging society. The emotional health of the elderly is gaining importance. Social trust is an important factor affecting emotional health, but existing studies have rarely considered the various effects of different types of social trust on rural elderly emotional health. Few studies have analysed the role of subjective well-being and subjective social status in the relationship between social trust and elderly emotional health.

**Methods:**

Using the data of the China Labor-force Dynamics Survey 2016 (CLDS 2016) and regression models, this study selected 2084 rural respondents aged 60 years and above to analyse the impact of social trust on their emotional health. Social trust was divided into three categories: trust in family members, trust in friends, and trust in neighbours. This study also examined the mediating and moderating effects of subjective well-being and subjective social status on the relationship between social trust and emotional health.

**Results:**

Trust in family members was significantly and positively associated with emotional health (coefficient = 0.194, *P* < 0.01) and subjective well-being (coefficient = 0.177, *P* < 0.01). Trust in friends was significantly and positively associated with emotional health and subjective well-being (coefficient = 0.097, *P* < 0.01; coefficient = 0.174, *P* < 0.01, respectively). Trust in neighbours was significantly and positively associated with emotional health and subjective well-being (coefficient = 0.088, *P* < 0.01; coefficient = 0.177, *P* < 0.01; respectively). Subjective well-being effectively reduced the impact of social trust in family, friends, and neighbours on the emotional health of the elderly by 0.023, 0.022, and 0.023, respectively. Trust in friends and neighbours significantly and positively affected respondents’ subjective social status (coefficient = 0.120, *P* < 0.05; coefficient = 0.090, *P* < 0.10; respectively). Subjective social status effectively reduced the impact of social trust in friends and neighbours on the emotional health of the elderly both by 0.004. The positive relationship between trust in family members and emotional health is weakened by subjective well-being.

**Conclusions:**

Social trust, especially family relationships, play an important role in maintaining the emotional health of the rural elderly. In response to population ageing, more social policies must be introduced to care for the rural elderly and help them lead a happy and satisfactory life.

## Background

Aging is an important social process that many developing countries are experiencing. China is the world’s most populous country, and its elderly population is growing [1]. By 2019, 18.1% of China’s population was over the age of 60 years. Over the past 40 years, China has experienced rapid urbanisation and modernisation, along with profound changes in people’s interpersonal relationships and lifestyles [2, 3]. However, compared to developed countries, China still has many deficiencies in elderly care and health welfare [4, 5]. The mental health of the elderly in particular has been neglected, and only a small number of older people seek help and treatment for psychological problems [6, 7]. Therefore, an examination of the mental health of the elderly and the factors influencing it is of great significance for improving their overall health and quality of life. The elderly living in rural areas would especially benefit from such research because they are affected by the huge gap between urban and rural public health services [2, 8].

The emotional health of the elderly is affected by many factors. In China, which is influenced by traditional family values, many older people live with their children and help in taking care of grandchildren and in family affairs [9, 10]. A good relationship with family members is, therefore, an important factor affecting elderly emotional health. For example, Tang et al. [11] found that Chinese older adults living with both a spouse and adult children reported better mental health than those living alone. Using a 1992 baseline survey of the Beijing Multidimensional Longitudinal Study on Aging, Chen and Silverstein [12] reported that providing instrumental support to children and satisfaction with children directly improved older Chinese parents’ well-being. In contrast, empty-nest elderly received less social support and were more likely to suffer from depression than those who lived with adult children [13–15]. Additionally, although social support has been shown to have a positive effect on maintaining the mental health of the elderly [16, 17], few studies have compared the effects of different types of social trust on the emotional health of the elderly. In China, most social interactions of the elderly are characterized by circle distribution, in which the core is the family life circle, followed by the circle of close friends, and the outermost circle of neighbours or strangers. Older people have different levels of social trust in different groups, which may have varying effects on their emotional health. Therefore, this study compared the differences in the impact of three types of social trust, namely trust in family, trust in friends, and trust in neighbours, on the emotional health of the elderly.

Studies have confirmed that subjective well-being and subjective social status are closely related to elders’ mental health [18, 19]. Subjective well-being and subjective social status reflect a person’s overall perception of life conditions, which are influenced by many factors, such as income and social trust [19–25]. A happy life may also have an important protective effect on the mental state of older people. Subjective social status has also been shown to be an important factor affecting emotional health [26, 27]. For instance, Hwang et al. [28] revealed that elderly individuals who reported a higher level of socioeconomic status were less likely to engage in risk behaviours, and were therefore more likely to have better health status. In this study, we considered subjective well-being and subjective social status as important moderating and mediating variables, and analysed the roles and effects of these two factors in the relationship between social trust and emotional health of the elderly in China.

**Fig. 1:**
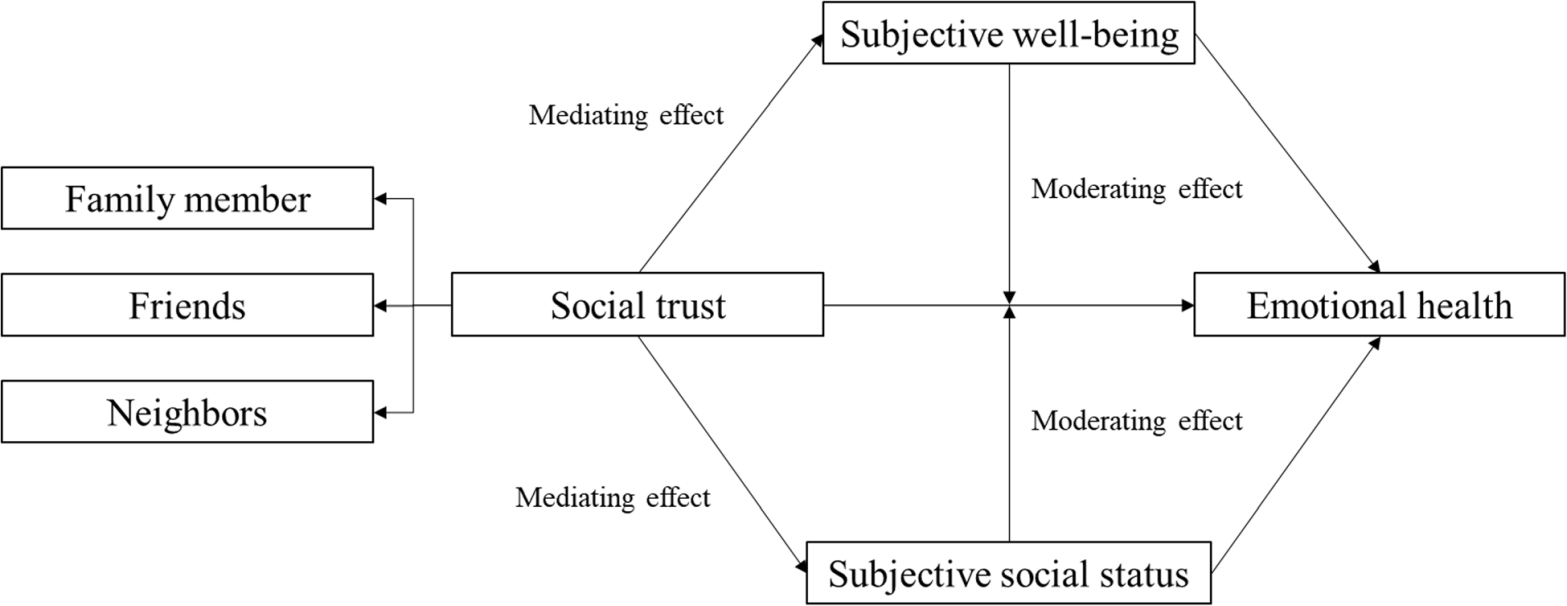
Research framework of this study

Based on the above analysis, we put forward the theoretical framework of this study (see Fig. 1). Firstly, in this study, we will distinguish the impact of social trust in different groups (family member vs. friends vs. neighbors). Social trust mainly reflects the degree of people’s interpersonal trust [29], which is directly influenced by the closeness of social relationships. In China’s rural society, the degree of closeness in interpersonal relationships presents a ‘differential pattern’ (cha xu ge ju) [30], with family members at the centre and different people in different positions in the social network. Generally, people’s social trust in family members may be higher than the social trust in friends and neighbors. Secondly, this study will analyse the impact of social trust on the emotional health of the elderly in rural China. There may be four influence paths of social trust on emotional health: (1) social trust → subjective well-being → emotional health; (2) social trust → subjective social status → emotional health; (3) subjective well-being plays a moderating role between social trust and emotional health; (4) subjective social status plays a moderating role between social trust and emotional health. We will examine those influence paths.

## Methods

### Data and sample

This study used data from the 2016 wave of the China Labor-force Dynamics Survey (CLDS 2016). The CLDS 2016 was conducted by the Center for Social Survey of Sun Yat-sen University. The respondents for this study were chosen from the CLDS 2016 data, by using a probability proportional to size sampling technique. As the target population of this study were the rural elderly in China, we selected a sample of respondents over the age of 60 year and living in rural neighbourhoods. After excluding respondents with missing information, the data of 2084 respondents were used in this study.

### Variables

#### Emotional health

Emotional health is a comprehensive expression of one’s inner world. In existing studies, there are two main ways to measure individuals’ emotional health. One is to focus on a certain aspect of emotional health, such as depression, and the other is to evaluate emotional health in general [31]. In this study, the measure of emotional health of rural elderly people was the respondents’ overall evaluation of their emotional health. Respondents were asked the question: ‘Did you have any emotional problems (such as feeling anxious or depressed) in the past month?’ The responses were recorded on a five-point scale (regrouped), wherein 5 = no, 4 = rarely, 3 = sometimes, 2 = frequently, and 1 = always; higher scores indicated better emotional health.

#### Social trust

Social trust is a state of the relationship between people and society [32]. In this study, social trust refers to the degree of trust the elderly have in other people with whom they interact frequently in daily life. Social trust was divided into three categories: trust in family members, trust in friends, and trust in neighbours. Social trust in each group was rated on a scale of 1 to 5 (1 = completely untrustworthy, 2 = relatively untrustworthy, 3 = between trustworthy and untrustworthy, 4 = relatively trustworthy, 5 = completely trustworthy), with a higher score indicating a higher level of trust in the particular group.

#### Subjective well-being and subjective social status

Subjective well-being and subjective social status were the main independent variables in this study. Subjective well-being is an individual’s overall evaluation of their life state [33]. Emotional health is closely related to personality and attitude [34], but it may also be affected by the individual’s life conditions (in this regard, we use subjective well-being as an indicator). The subjective social status is obtained by comparing the economic and social conditions of individuals with other people, which is a subjective cognition that is obtained relative to others [35]. In this research, subjective well-being is obtained by directly asking the respondent ‘In general, do you think you are living a happy life?’ Subjective well-being was measured through the respondents’ evaluation of their happiness rated from 1 to 5 (1 = very unhappy, 2 = unhappy, 3 = average, 4 = happy, 5 = very happy), with higher scores indicating higher subjective well-being. The MacArthur Scale of subjective social status was used to assesses the respondents’ perceived rank relative to others in their group, which is widely used in previous studies [36, 37]. Subjective social status was assessed on a 10-rung ladder measure of social class rank (a self-anchoring scale) ranging from 1 to 10, with higher scores indicating higher subjective social status.

#### Control variables

Individual demographic factors were used as control variables. The control variables were: age (continuous variable), marital status (categorical variable), number of family members living together (continuous variable), work status (categorical variable), (log) annual personal income (continuous variable), gender (categorical variable), educational level (continuous variable), and self-rated health (continuous variable). Self-rated health data were obtained by asking respondents about their physical health, rated on a scale from 1 (very healthy) to 5 (very unhealthy), with higher scores indicating lower self-rated health.

### Methods description

Emotional health (the independent variable) is continuous variable in this study. Therefore, we use linear regression method to detect the influence of social trust on emotional health. In the analysis of the mediation effect, we use the Baron and Kenny [38] method to test the mediation hypotheses. In this study, the mediators include subjective well-being and subjective social status. We test the following influence paths: (1) social trust (trust in family members, trust in friends, and trust in neighbors) to subjective well-being to emotional health; (2) social trust (trust in family members, trust in friends, and trust in neighbors) to subjective social status to emotional health. In terms of moderation analysis, we adopt interaction methods to measure the moderation effect of subjective well-being (moderator) and subjective social status (moderator).

## Results

### Mediation effects of subjective well-being and subjective social status on the relationship between social trust and emotional health of older adults

Table 1 presents the descriptive statistics of all the variables used in the analysis. In general, the average level of emotional health of elderly respondents was relatively high, at 4.22. Among the three types of social trust, trust in family members was the highest (4.82), followed by trust in friends (4.35), and the lowest was trust in neighbours (3.95). The average subjective well-being of elderly respondents was 3.77. The subjective social status of the elderly was relatively low, with an average of 4.24 (< 5). In terms of the respondents’ demographic characteristics, the average age of the respondents was 65.23, and most of the elderly had partners (92.08%). In terms of family composition, the average number of family members living together was 5.05. In terms of working status, 94.24% of the elderly respondents had a job (mostly working in agriculture). The average annual income of the respondents was 11,889.3 yuan. The proportions of male respondents and female respondents were 61.13 and 38.87%, respectively. The average education level of the respondents was 2.04, which is low. The respondents’ average self-rated health was 2.79.

**Table 1:** Descriptive statistics of the variables used in the analysis (*n* = 2084)

Variables	Mean (SD)/Percentage
Emotional health (1–5)	4.22 (SD = 1.02)
Trust in family members (1–5)	4.82 (SD = 0.48)
Trust in friends (1–5)	4.35 (S = 0.71)
Trust in neighbors (1–5)	3.95 (SD = 0.79)
subjective well-being (1–5)	3.77 (SD = 0.90)
Subjective social status (1–10)	4.24 (SD = 1.76)
Age (> = 60 years old)	65.23 (SD = 1.76)
Marital status (%)	
Single	0.91
Married or cohabit	92.08
Widowed or divorced	7.01
Number of family members living together	5.05 (SD = 2.52)
Work status (%)	
In work	94.24
Out of work	5.76
Annual personal income (yuan)	11,889.3 (SD = 19,037.3)
Gender (%)	
Male	61.13
Female	38.87
Educational level (1–9)	2.04 (SD = 1.01)
Self-rated health (1–5)	2.79 (SD = 1.02)

Table 2 shows the regression results on the relationship of social trust and emotional health and the mediation effect of subjective well-being. The dependent variable in Models 1, 3, 4, 6, 7, and 9 was emotional health, and the dependent variable in Models 2, 5, and 8 was subjective well-being. Model 1 shows that trust in family members is significantly and positively associated with respondents’ emotional health (coefficient = 0.194, *P* < 0.01). Model 2 shows that trust in family members had a significant positive effect on the subjective well-being of the respondents (coefficient = 0.177, *P* < 0.01). Models 4 and 5 show that trust in friends was significantly and positively associated with respondents’ emotional health and their subjective well-being (coefficient = 0.097, *P* < 0.01; coefficient = 0.174, *P* < 0.01; respectively). Models 7 and 8 show that trust in neighbours was significantly and positively associated with respondents’ emotional health and their subjective well-being (coefficient = 0.088, *P* < 0.01; coefficient = 0.177, *P* < 0.01; respectively).

**Table 2:** The relationship of social trust and emotional health and the mediation effects of subjective well-being

	Trust in family members						Trust in friends						Trust in neighbors					
	Model 1: emotional health		Model 2: subjective well-being		Model 3: emotional health		Model 4: emotional health		Model 5: subjective well-being		Model 6: emotional health		Model 7: emotional health		Model 8: subjective well-being		Model 9: emotional health	
	Coefficient	S.E.	Coefficient	S.E.	Coefficient	S.E.	Coefficient	S.E.	Coefficient	S.E.	Coefficient	S.E.	Coefficient	S.E.	Coefficient	S.E.	Coefficient	S.E.
Trust in family members	0.194***	(0.043)	0.177***	(0.040)	0.171***	(0.043)												
Trust in friends							0.097***	(0.030)	0.174***	(0.027)	0.075**	(0.030)						
Trust in neighbors													0.088***	(0.027)	0.177***	(0.024)	0.065**	(0.027)
subjective well-being					0.131***	(0.024)					0.132***	(0.024)					0.131***	(0.024)
Age	0.005	(0.005)	0.017***	(0.004)	0.002	(0.005)	0.005	(0.005)	0.017***	(0.004)	0.003	(0.005)	0.005	(0.005)	0.017***	(0.004)	0.002	(0.005)
Marital status (ref: single)																		
married or cohabit	0.093	(0.220)	0.234	(0.201)	0.063	(0.219)	0.078	(0.221)	0.198	(0.200)	0.052	(0.219)	0.083	(0.221)	0.202	(0.200)	0.057	(0.219)
widowed or divorced	0.063	(0.232)	0.094	(0.212)	0.051	(0.231)	0.042	(0.233)	0.053	(0.211)	0.035	(0.231)	0.040	(0.233)	0.043	(0.211)	0.034	(0.231)
Number of family members living together	−0.006	(0.008)	−0.011	(0.008)	−0.005	(0.008)	−0.006	(0.008)	−0.009	(0.008)	−0.004	(0.008)	−0.006	(0.008)	−0.008	(0.008)	−0.005	(0.008)
work status (ref: in work)	− 0.068	(0.090)	− 0.015	(0.082)	− 0.066	(0.089)	− 0.082	(0.090)	− 0.036	(0.082)	−0.077	(0.090)	−0.078	(0.090)	−0.030	(0.082)	−0.074	(0.090)
(log) annual personal income	0.003	(0.008)	0.015**	(0.007)	0.001	(0.008)	0.002	(0.008)	0.014**	(0.007)	0.000	(0.008)	0.002	(0.008)	0.014**	(0.007)	0.000	(0.008)
Female (ref: male)	−0.215***	(0.047)	0.037	(0.043)	−0.220***	(0.047)	−0.208***	(0.047)	0.044	(0.043)	−0.214***	(0.047)	−0.206***	(0.047)	0.048	(0.043)	−0.213***	(0.047)
educational level	0.045**	(0.023)	0.035*	(0.021)	0.041*	(0.022)	0.045**	(0.023)	0.035*	(0.020)	0.041*	(0.022)	0.045**	(0.023)	0.035*	(0.020)	0.040*	(0.022)
Self-rated health	−0.298***	(0.021)	−0.191***	(0.019)	−0.273***	(0.021)	−0.296***	(0.021)	−0.180***	(0.019)	−0.273***	(0.021)	−0.298***	(0.021)	−0.181***	(0.019)	−0.274***	(0.021)
_cons	3.729***	(0.443)	1.953***	(0.406)	3.474***	(0.443)	4.239***	(0.413)	2.038***	(0.375)	3.971***	(0.413)	4.319***	(0.406)	2.097***	(0.367)	4.045***	(0.406)
N	2084		2084		2084		2084		2084		2084		2084		2084		2084	
R-sq	0.132		0.076		0.144		0.128		0.085		0.140		0.128		0.090		0.140	
adj. R-sq	0.127		0.071		0.140		0.123		0.080		0.136		0.124		0.086		0.135	
Log lik.	− 2840.335		− 2654.713		− 2825.309		− 2845.088		− 2644.319		− 2830.070		−2845.022		− 2638.266		−2830.282	

Coefficient is unstandardized. Standard errors in parentheses. * *p* < 0.10, ** *p* < 0.05, *** *p* < 0.01

**Fig. 2:**
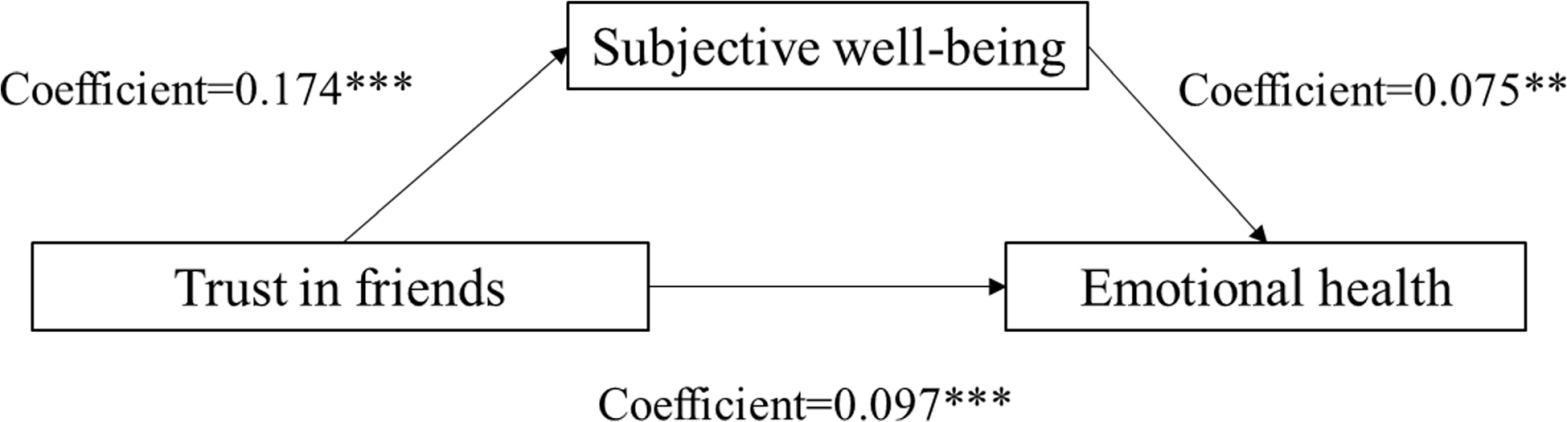
Unstandardized coefficients estimating trust in family members→subjective well-being→mental health; * = *p* < .10, ** = *p* < .05, *** = *p* < .01

**Fig. 3:**
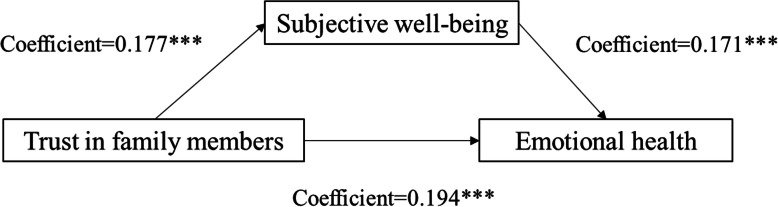
Unstandardized coefficients estimating trust in friends→subjective well-being→ mental health; * = *p* < .10, ** = *p* < .05, *** = *p* < .01

**Fig. 4:**
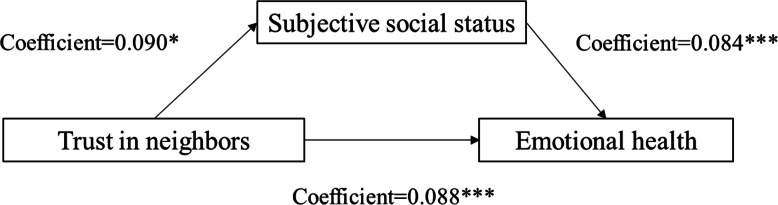
Unstandardized coefficients estimating trust in neighbors→subjective well-being→ mental health; * = *p* < .10, ** = *p* < .05, *** = *p* < .01

We followed Baron and Kenny [38] to test the presence of mediation effects of subjective well-being. Table 3 shows the direct and indirect effects of social trust on the emotional health of the elderly under the mediational effect of subjective well-being and subjective social status. The results show that there are 3 significant influence paths from social trust to emotional health through subjective well-being. Figs. 2, 3 and 4 show the mediation effect for subjective well-being between trust in family members and emotional health (coefficient of direct effect = 0.171; coefficient of indirect effect = 0.023), between trust in friends and emotional health (coefficient of direct effect = 0.075; coefficient of indirect effect = 0.022), between trust in neighbors and emotional health (coefficient of direct effect = 0.088; coefficient of indirect effect = 0.023), respectively. These results suggest that subjective well-being can effectively reduce the impact of social trust (in family, friends, and neighbours) on the emotional health of rural older respondents.

**Table 3:** Summary of direct and indirect effects of social trust on emotional health

Mediator	Path	Direct effect	Indirect effect
Subjective well-being	Trust in family members → subjective well-being → emotional health	0.171	0.023
	Trust in friends → subjective well-being → emotional health	0.075	0.022
	Trust in neighbors → subjective well-being → emotional health	0.088	0.023
Subjective social status	Trust in friends → subjective social status → emotional health	0.093	0.004
	Trust in neighbors → subjective social status → emotional health	0.084	0.004

Table 4 shows the regression results of the mediation effect of subjective social status on the relationship between social trust and emotional health. Model 10 shows that trust in family members had no significant effect on respondents’ subjective social status. Model 12 and 14 show that trust in friends and trust in neighbours had significant positive effects on respondents’ subjective social status (coefficient = 0.120, *P* < 0.05; coefficient = 0.090, *P* < 0.10; respectively).

**Table 4:** The regression results of mediation effects of subjective social status on the relationship between social trust and emotional health

	Trust in family members						Trust in friends						Trust in neighbors					
	Model 1: emotional health		Model 10: subjective social status		Model 11: emotional health		Model 4: emotional health		Model 12: subjective social status		Model 13: emotional health		Model 7: emotional health		Model 14: subjective social status		Model 15: emotional health	
	Coefficient	S.E.	Coefficient	S.E.	Coefficient	S.E.	Coefficient	S.E.	Coefficient	S.E.	Coefficient	S.E.	Coefficient	S.E.	Coefficient	S.E.	Coefficient	S.E.
Trust in family members	0.194***	(0.043)	0.118	(0.078)	0.190***	(0.043)												
Trust in friends							0.097***	(0.030)	0.120**	(0.054)	0.093***	(0.030)						
Trust in neighbors													0.088***	(0.027)	0.090*	(0.048)	0.084***	(0.027)
subjective social status					0.037***	(0.012)					0.037***	(0.012)					0.037***	(0.012)
Age	0.005	(0.005)	0.022***	(0.008)	0.004	(0.005)	0.005	(0.005)	0.022***	(0.008)	0.004	(0.005)	0.005	(0.005)	0.022***	(0.008)	0.004	(0.005)
marital status (ref: single)																		
married or cohabit	0.093	(0.220)	0.845**	(0.399)	0.062	(0.220)	0.078	(0.221)	0.820**	(0.399)	0.048	(0.220)	0.083	(0.221)	0.830**	(0.399)	0.052	(0.220)
widowed or divorced	0.063	(0.232)	0.685	(0.421)	0.038	(0.232)	0.042	(0.233)	0.657	(0.421)	0.018	(0.233)	0.040	(0.233)	0.660	(0.421)	0.016	(0.233)
Number of family members living together	−0.006	(0.008)	0.005	(0.015)	−0.006	(0.008)	−0.006	(0.008)	0.007	(0.015)	−0.006	(0.008)	−0.006	(0.008)	0.006	(0.015)	−0.006	(0.008)
work status (ref: in work)	− 0.068	(0.090)	0.151	(0.163)	−0.073	(0.090)	−0.082	(0.090)	0.137	(0.163)	−0.087	(0.090)	−0.078	(0.090)	0.142	(0.163)	−0.083	(0.090)
(log) annual personal income	0.003	(0.008)	0.034**	(0.014)	0.001	(0.008)	0.002	(0.008)	0.034**	(0.014)	0.001	(0.008)	0.002	(0.008)	0.034**	(0.014)	0.001	(0.008)
Female (ref: male)	−0.215***	(0.047)	−0.020	(0.086)	−0.214***	(0.047)	−0.208***	(0.047)	−0.015	(0.086)	−0.208***	(0.047)	−0.206***	(0.047)	−0.013	(0.086)	−0.206***	(0.047)
educational level	0.045**	(0.023)	0.111***	(0.041)	0.041*	(0.023)	0.045**	(0.023)	0.111***	(0.041)	0.041*	(0.023)	0.045**	(0.023)	0.110***	(0.041)	0.041*	(0.023)
Self-rated health	−0.298***	(0.021)	−0.290***	(0.038)	−0.288***	(0.021)	−0.296***	(0.021)	−0.282***	(0.038)	−0.286***	(0.021)	−0.298***	(0.021)	−0.286***	(0.038)	−0.287***	(0.021)
_cons	3.729***	(0.443)	1.708**	(0.804)	3.667***	(0.443)	4.239***	(0.413)	1.745**	(0.747)	4.176***	(0.413)	4.319***	(0.406)	1.916***	(0.734)	4.249***	(0.406)
N	2084		2084		2084		2084		2084		2084		2084		2084		2084	
R-sq	0.132		0.048		0.135		0.128		0.049		0.131		0.128		0.049		0.132	
adj. R-sq	0.127		0.043		0.131		0.123		0.045		0.127		0.124		0.044		0.127	
Log lik.	−2840.335		− 4081.161		− 2835.724		−2845.088		− 4079.822		−2840.526		−2845.022		− 4080.557		−2840.388	

Coefficient is unstandardized. Standard errors in parentheses. * *p* < 0.10, ** *p* < 0.05, *** *p* < 0.01

**Fig. 5:**
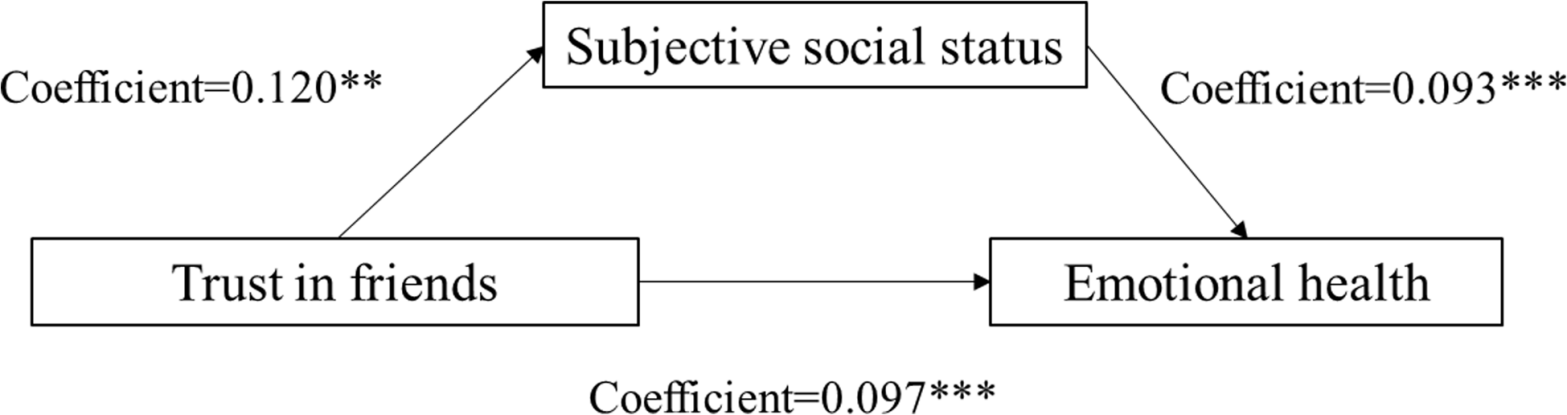
Unstandardized coefficients estimating trust in friends→ subjective social status→ mental health; * = *p* < .10, ** = *p* < .05, *** = *p* < .01

**Fig. 6:**
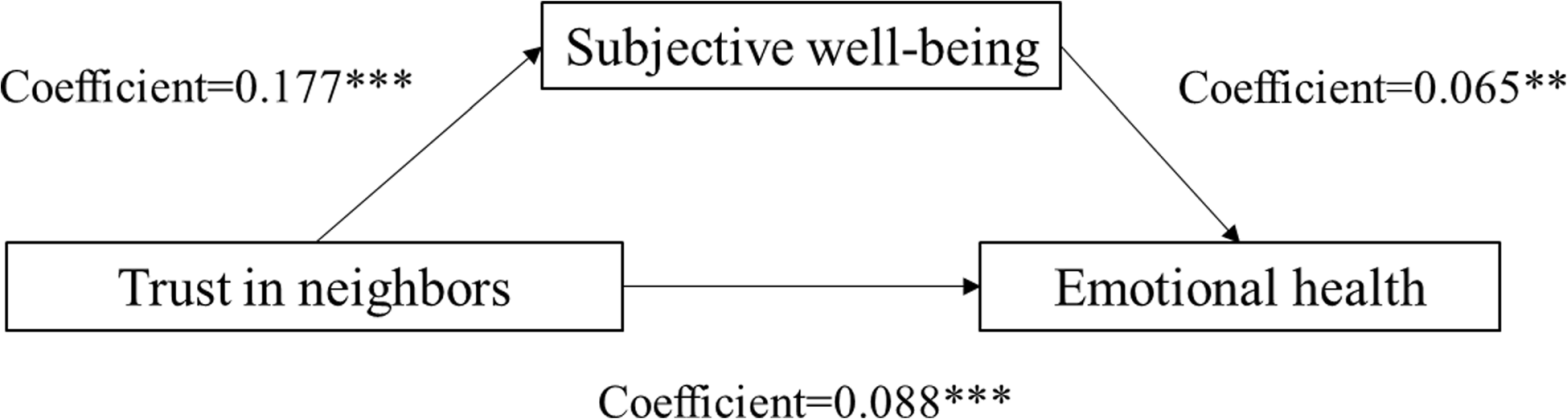
Unstandardized coefficients estimating trust in neighbors→ subjective social status→mental health; * = *p* < .10, ** = *p* < .05, *** = *p* < .01

We also followed Baron and Kenny [38] to test the presence of mediation effects of subjective social status. The results show that there are 2 significant influence paths from social trust to emotional health through subjective social status. Figs. 5 and 6 show the mediation effect for subjective social status between trust in friends and emotional health (coefficient of direct effect = 0.093; coefficient of indirect effect = 0.004), between trust in neighbors and emotional health (coefficient of direct effect = 0.084; coefficient of indirect effect = 0.004), respectively. These results suggest that the mediating role of subjective social status in the relationship between social trust and emotional health is very small.

### Moderation effects of subjective well-being and subjective social status on the relationship between social trust and emotional health of older adults

**Fig. 7:**
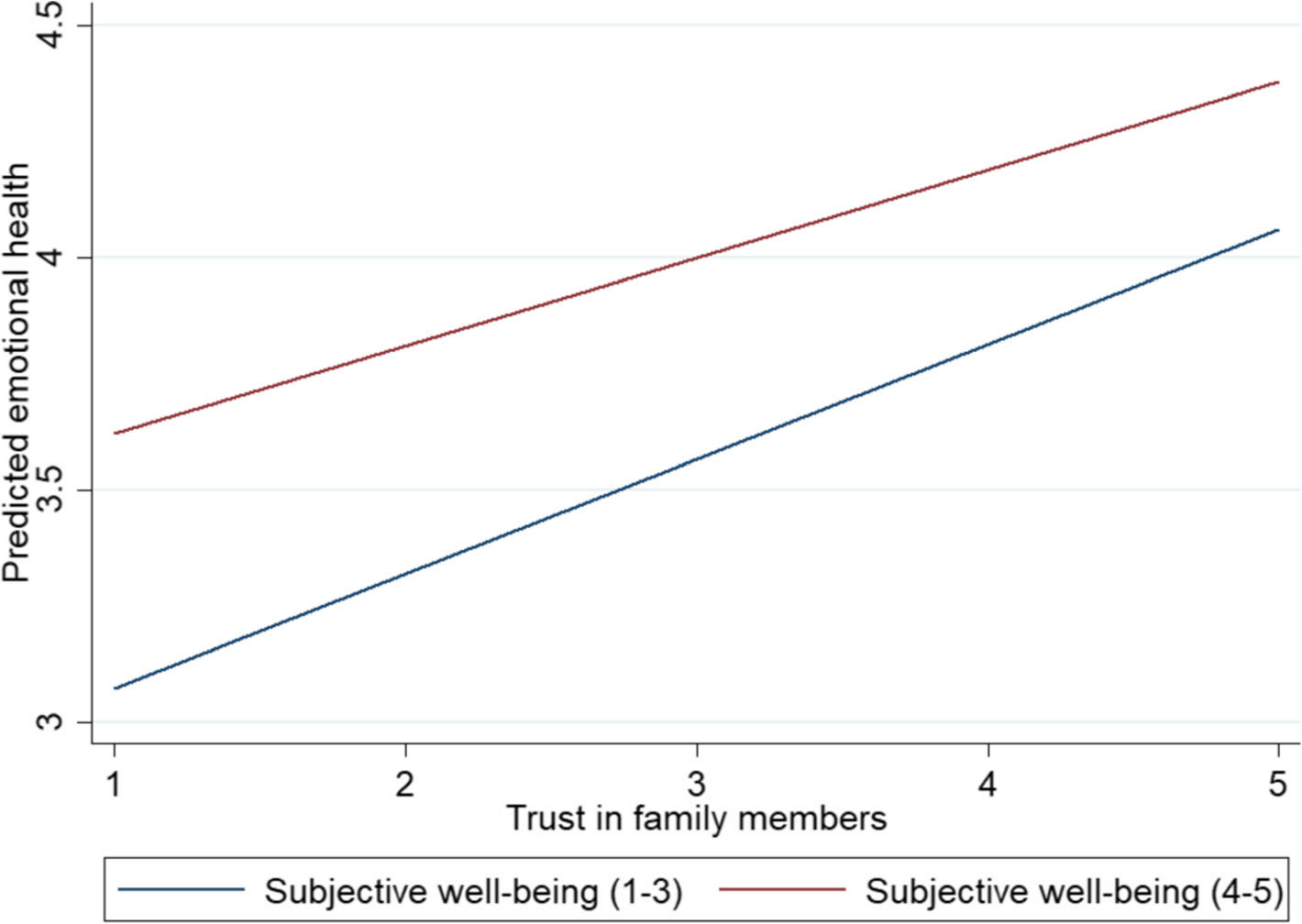
The predicated relationship between trust in family members and emotional health differing by subjective well-being

**Fig. 8:**
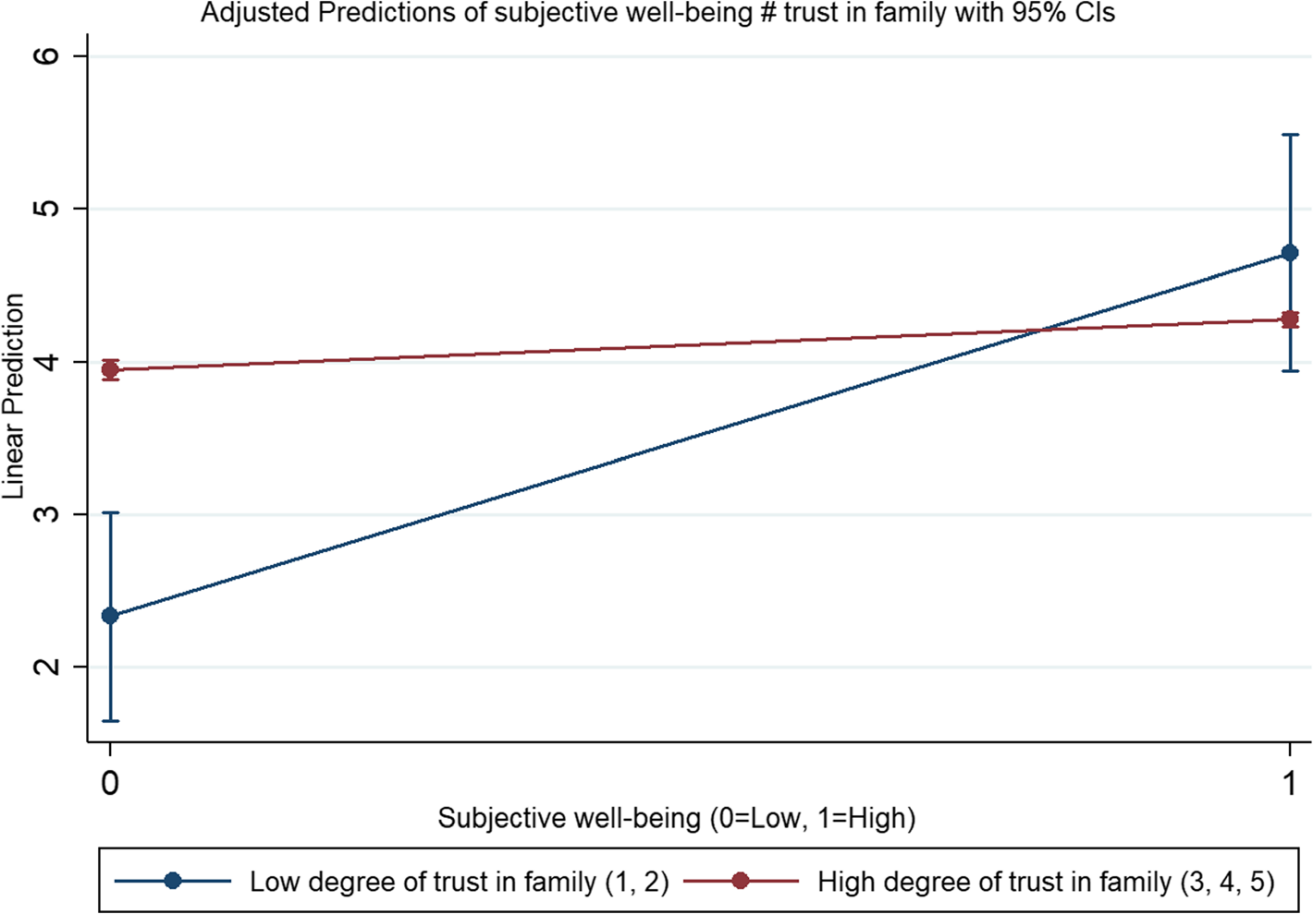
Moderating effects of subjective well-being on the relationship between trust in family members and emotional health

Table 5 shows the regression results of the moderation effects of subjective well-being and subjective social status on the relationship between social trust and emotional health. The results from Model 16 show that both subjective well-being and subjective social status have significant effect on rural older respondents’ emotional health. Model 17 and Figs. 7 and 8 show that the positive relationship between trust in family members and emotional health was weakened by subjective well-being, but subjective social status had no moderating effect on the relationship between social trust and emotional health.

**Table 5:** The regression results of moderation effects of subjective well-being and subjective social status on the relationship between social trust and emotional health

	Model 16: emotional health		Model 17: emotional health	
	Coefficient	S.E.	Coefficient	S.E.
Subjective well-being	0.115***	(0.025)	0.806***	(0.188)
Subjective social status	0.021*	(0.012)	0.064	(0.128)
Trust in family members	0.152***	(0.047)	0.369**	(0.166)
Trust in friends	0.007	(0.040)	0.134	(0.166)
Trust in neighbors	0.037	(0.034)	0.287**	(0.140)
Age	0.002	(0.005)	0.003	(0.005)
marital status (ref: single)				
married or cohabit	0.039	(0.219)	0.046	(0.218)
widowed or divorced	0.026	(0.231)	0.032	(0.230)
Number of family members living together	−0.004	(0.008)	−0.003	(0.008)
work status (ref: in work)	−0.073	(0.089)	−0.069	(0.089)
(log) annual personal income	0.000 (0.00002)	(0.008)	−0.000 (− 0.0003)	(0.008)
Female (ref: male)	−0.217***	(0.047)	−0.205***	(0.047)
educational level	0.039*	(0.022)	0.038*	(0.022)
Self-rated health	−0.267***	(0.022)	−0.265***	(0.021)
Interactions				
Trust in family members # Subjective well-being			−0.087**	(0.043)
Trust in friends # Subjective well-being			−0.000 (− 0.00003)	(0.044)
Trust in neighbors # Subjective well-being			−0.072*	(0.037)
Trust in family members # Subjective social status			0.018	(0.027)
Trust in friends # Subjective social status			−0.034	(0.023)
Trust in neighbors # Subjective social status			0.005	(0.019)
_cons	3.386***	(0.446)	0.793	(0.844)
N	2084		2084	
R-sq	0.146		0.155	
adj. R-sq	0.140		0.147	
Log lik.	− 2822.860		− 2811.344	

Coefficient is unstandardized. Standard errors in parentheses. * *p* < 0.10, ** *p* < 0.05, *** *p* < 0.01

## Discussion

Few previous studies have compared the effects of different types of social trust on the mental health of the rural elderly [39, 40]. The present findings fill this research gap. We analysed the effects of social trust on the emotional health of the rural elderly, including their trust in family, friends, and neighbours. The results indicate that trust in family had the greatest impact on the emotional health of the elderly, followed by trust in friends, and then, trust in neighbours. This result can be considered as a ‘differential pattern’ (cha xu ge ju) [41] in the emotional health of the elderly in rural China. This is a pattern of difference in the relationship between social trust and emotional health, in which family members provide the greatest emotional support to the elderly in rural China. Although the rapid economic development and modernisation process in the past few decades have had a profound impact on the social structure of China [42], for those over 60 years of age, the emotional support of traditional family relationships remains strong [43]. A family member’s support has important implications for the mental health of older persons.

Subjective well-being and subjective social status are not only psychological states, but also have positive emotional health effects [44, 45]. This study found that subjective well-being and subjective social status have a certain protective effect on the emotional health of the rural elderly in China. Both can effectively reduce the degree of the impact of social trust on the emotional health of the rural elderly. Subjective well-being and subjective social status are the overall cognitive evaluation of one’s quality of life. Older people with higher degrees of happiness and social status have a stronger ability to cope with mental health risks, and they may have a stronger ability to make social interactions less likely to affect their emotional health. A higher quality of life can also enable older people to overcome depression more quickly. In addition, social status is related to social capital or social resources that one has or can use [46]. Older people with higher subjective social status may have more social resources to mitigate the impact of external factors on their emotional health. In China, the happiness and socio-economic status of the rural elderly will continue to decline with increasing age and the deterioration of their physical health. What is concerning is that the subjective social status of the elderly in rural China is generally low. The rural elderly who think they are at the bottom of society may have more serious emotional problems when they encounter a crisis of trust. Giving the elderly a higher social status and quality of life is very important for their life quality and emotional health.

China is fast becoming an aging society; with the huge urban-rural development gap [47], the rural elderly group has become disadvantaged as it has been neglected for a long time. The protection of the mental health of the rural elderly population has become increasingly important. Based on the above findings, we propose policy recommendations in the following four aspects. First, only by giving the rural elderly the opportunity to continue to contribute to society can they maintain their social status in society. It is increasingly urgent to improve the well-being and social status of the elderly. When they retire or return from the city to the countryside after losing labour owing to age, most rural elderly is left without the channels and opportunities that can help them continue developing their talents. As the number of elderly people continues to increase, China must accelerate the development of the silver economy, and encourage elderly people to find suitable jobs and ensure the quality of life. Second, maintaining harmonious family relationships has an important impact on the emotional health of the rural elderly. Faced with the increasing generational separation of family members [48], the Chinese society should advocate the integrity of the family (especially urban-rural migrant families that have been separated [24]), so that the rural elderly could get emotional support from their family members immediately when they may need it. Third, the Chinese government should urgently address the mental health of the rural elderly and improve the social welfare system in rural China. In China, the lifestyle of many elderly people is very monotonous, and there is no opportunity for them to work or continue to meaningfully participate in society after retirement [49, 50]. It is crucial to provide the elderly with more opportunities to participate in social activities in order to alleviate elderly depression. Fourth, community mental health services are a shortcoming of China’s health services. It is necessary to establish a community-based mental health service system to allow more elderly people to receive formal and direct mental health support.

In terms of research limitations, first, owing to limited data, this study did not compare and analyse the emotional health of the rural elderly population of different age groups, but there may be some differences in the emotional health and the factors affecting emotional health of elderly residents of different age groups (middle-aged, elderly, and elderly). Second, although in this study the number of family members living together did not have a significant impact on the emotional health of the elderly, for the elderly in China, the family is still a very important source of emotional support. For example, in intergenerational families, a harmonious relationship between grandparents and grandchildren can provide important emotional support to the elderly [24]. Third, there are significant gender differences in terms of the emotional health of the rural elderly. Gender equality is a major social issue faced by China, especially in case of the rural elderly. The health needs and social status protection of rural elderly people belonging to different genders warrant further research.

## Conclusion

This study found that trust in family members, friends, and neighbours each has a significant positive effect on the emotional health, subjective well-being, and social status of the rural elderly in China. Subjective well-being and subjective social status play a significant mediating role in the relationship between social trust and the emotional health of the rural elderly. The positive relationship between trust in family members and emotional health is weakened by subjective well-being, but subjective social status has no moderating effect on the relationship between social trust and emotional health of the rural elderly. This study suggests that family relationships play an important role in maintaining the emotional health of the elderly in rural China. In response to the increase in the aging population in China, social policies to care for the rural elderly and help them live a happy and decent life must be urgently introduced.

## Data Availability

Data used in this study were derived from the 2016 wave of the China Labor-force Dynamics Survey (CLDS 2016), which was conducted by the Center for Social Survey of Sun Yat-sen University. Public access to the database is open. The datasets generated and/or analysed during the current study are available in the Center for Social Survey of Sun Yat-sen University repository [cssdata@mail.sysu.edu.cn]. The opinions in this paper are those of the authors.

## Abbreviations

CLDS: China Labor-force Dynamics Survey
CES-D: Center for Epidemiologic Studies Depression Scale
